# In the spotlight—Established researcher

**DOI:** 10.1002/jez.b.23170

**Published:** 2022-07-26

**Authors:** Marcelo R. Sánchez‐Villagra

**Affiliations:** ^1^ Paleontological Institute and Museum University of Zurich Zurich Switzerland

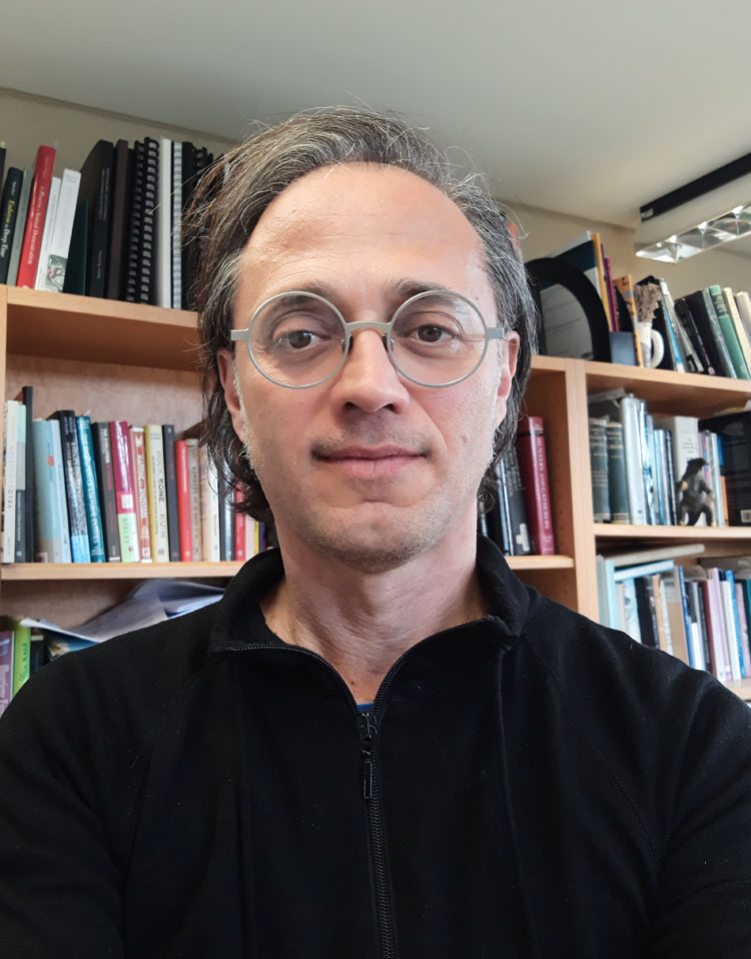

Marcelo Sánchez‐Villagra is Guest‐Editor of this special issue on the evolutionary developmental morphology of domestication.

Website: www.msanchezlab.net

Google scholar page: https://scholar.google.com/citations?user=taTQzw0AAAAJ

***With whom and where did you study?***

My undergraduate study in Biology was at Universidad Simón Bolívar in Caracas. After a year of fieldwork and diverse laboratory experiences, I went for a PhD at Duke University, with a thesis on marsupial mammal cranial development and evolution. I had two coadvisors: Kathleen Smith (comparative ontogenetics) and Richard Kay (paleontological work). This was followed by my Habilitation under my mentor Wolfgang Maier in Tübingen (Germany), where I worked on diverse topics of mammalian ontogeny and learned to teach on the comparative anatomy of diverse Deuterostomia groups. During my job at the Natural History Museum in London, I learned about modularity from hosting Anjali Goswami as a postdoc; from many paleontologists there and in Zurich I was inspired to contribute to “developmental paleontology.”

***What got you interested in biology? When did you know EvoDevo was for you?***

I came to Biology with a fascination for exploring the natural world; evolution provided an explanation to my questions on origins. My first interest was in reconstructing evolutionary trees, and for that solving homology questions required the ontogenetic perspective.

Exposure to EvoDevo ideas came from readings at graduate school at Duke on the neural crest, heterochrony, evolutionary novelties, and others—there I learned that EvoDevo was not just about Hox genes, and I became inspired by Pere Alberch's papers. I started to use the sequence heterochrony approach following the work of Kathleen Smith, Mike Richardson, and others, as this allowed me to examine developmental evolution with a comparative approach that did not require perfectly timed series and thus could be more inclusive in taxonomic sampling. When I learned about palaeohistology from my then postdoc Torsten Scheyer in Zurich, I realized that one could directly address matters of growth and life history in fossils, in addition to an approach based on phylogenetic bracket considerations. For my work on animal domestication, I saw the chance to bring a comparative ontogenetic perspective, and here the insights gained on neural crest development by detailed experimental studies in the work of Rich Schneider and others inform much of what we discussed about patterns of morphological diversification.

***What do you see as the major challenges of EvoDevo?***

I hope that EvoDevo embraces genuinely comparative ontogenetic research as a part of it, and that technological advances continue to contribute with discoveries but do not determine what can be funded or published, as EvoDevo remains a question‐driven discipline as opposed to one driven by methods. Macroevolutionary questions that can be addressed only from a developmental perspective should continue to be part of a broad and pluralistic EvoDevo, as well as the explanation of phenotypic variation among populations within a species. I imagine that the examination of neglected groups of organisms or of organ systems will provide unanticipated insights on the amazing variation in developmental evolution.

It will be a challenge for the EvoDevo community to be inclusive in that it can be practiced by people across the world given the differential access to resources. Maybe some of the research in Eco‐EvoDevo will serve to better understand environmental issues faced by humanity, but I suspect it is more likely that EvoDevo will be more about satisfying human intellectual curiosity.

